
JOURNAL INFORMATION
==============================
NLM Title Abbreviation: Behav Neurol
Journal ID: Behav Neurol
NLM Journal ID: 8914585
Journal ID: BN

Behavioural Neurology

ISSN: 0953-4180
EISSN: 1875-8584
Publisher: Wiley

ARTICLE INFORMATION
==============================
PMCID: PMC5378046
PMID: 22063814
DOI: 10.3233/BEN-2011-0336
Article ID: 689675
Article version: 1
Subjects: Introduction

Language, Memory and the Temporal Lobes

Manes Facundo 1 2 3 *
Gleichgerrcht Ezequiel 1 3
1 Institute of Cognitive Neurology (INECO) Buenos Aires Argentina
2 Laboratory of Cognitive Neuroscience Universidad Diego Portales Santiago Chile
3 Institute of Neurosciences Favaloro University Buenos Aires Argentina
*Facundo Manes: fmanes@ineco.org.ar
Print publication date: 2011
Electronic publication date: 2011 Nov 7
Volume: 24
Issue: 4
First page: 263
Last page: 264
Received 2011 Oct 31; Accepted 2011 Oct 31
Copyright: Copyright © 2011 Hindawi Publishing Corporation and the authors.
Copyright year: 2011
License: This is an open access article distributed under the Creative Commons Attribution License, which permits unrestricted use, distribution, and reproduction in any medium, provided the original work is properly cited.
License URL: https://creativecommons.org/licenses/by/3.0/

==============================
Behavioural Neurology 24 (2011) 263–264
DOI 10.3233/BEN-2011-0336
IOS Press

### Introduction

### Language, Memory and the Temporal Lobes

Facundo Manes[a,b,c,*] and Ezequiel Gleichgerrcht[a,c]
[a]*Institute of Cognitive Neurology (INECO), Buenos Aires, Argentina*
[b]*Laboratory of Cognitive Neuroscience, Universidad Diego Portales, Santiago, Chile*
[c]*Institute of Neurosciences, Favaloro University, Buenos Aires, Argentina*

In recent decades, extensive data have been generated about the normal and pathological functioning of the temporal lobes, and their relationship with speech and semantics. Advancement in this field has contributed to the development of new approaches not only for the assessment of cognitive deficits associated with lesions and degeneration of the temporal lobe, but also for the development of novel techniques that may prove useful in the rehabilitation of language skills. This issue of *Behavioural Neurology* is dedicated to original research in the field of aphasia and the temporal lobes which was presented during the World Federation of Neurology's Research Group on Aphasia and Cognitive Disorders Meeting held during mid-May 2010 in Istanbul, Turkey. The articles in this issue survey many of these aspects and, taken together, demonstrate the interdisciplinary approach from which the field will benefit the most.

Sebastian Crutch and Elizabeth Warrington build on the phenomenon they refer to as "refractory semantic access dysphasia" by investigating the semantic organization associated with proper names in two global aphasic patients. They delve into the results of cognitive performance in these patients to contribute to the literature on taxonomy and theme-centered organization of proper names. In this same issue, Carlo Semenza critically reviews findings on naming with proper names (mostly person names) and discusses the relevance of the left temporal pole in this respect. Semenza's review provides insight into the discussion of the brain substrates underlying proper name processing and offers also some speculations as to how the brain has evolved to specifically support the development and retrieval of proper names.

Steven Rapcsak and Emily Edmonds review the functional impairment in face memory that results from frontal and temporal lobe lesions. They propose a model for facial memory processing that requires dynamic interactions between the executive controlled processes and a set of automatic processes that strongly depend on the temporal lobes.

Two articles in this issue are devoted to exploring the relationship between cardiovascular events and temporal lobe functioning. Chris Butler and Adam Zeman investigate the cardiovascular correlates of transient epileptic amnesia, which has been classically associated with temporal lobe epilepsy. Their study constitutes an important contribution to the current literature, as the heart-brain link is one that will result in the design of fundamental strategies for the prevention and treatment of several neurological conditions. Valentina La Corte and collaborators report lesions in sites not previously linked with confabulation, resulting from rupture of an anterior communicating artery aneurism. The unique characteristics of their case provide further details into particular aspects of the mechanisms underlying confabulations.

Anna Basso and Margherita Macis review the state-of-the art of the efficacy of aphasia therapy in chronic patients and report on the effects of intensive home-therapy for a large group of patients with severe chronic aphasic. Their results provide important information that can be readily translatable into specific strategies for treatment of severely aphasic chronic patients.

All articles included in this issue have a clear common denominator: their findings have relevant theoret-

*Address for correspondence. E-mail: fmanes@ineco.org.ar.

ISSN 0953-4180/11/$27.50 © 2011 – IOS Press and the authors. All rights reserved ical and clinical implications which, incorporated into the scientific literature and embraced by different aspects of patient care, will contribute to the advancement of this growing field. By doing so, we will potentiate the efforts put thus far in offering a better quality of life to patients who are affected by aphasia and other disorders associated with the temporal lobes.
